# Type 1 Diabetes Mellitus and Alexithymia: A Systematic Review

**DOI:** 10.3390/healthcare13192402

**Published:** 2025-09-24

**Authors:** Emanuele Maria Merlo, Liam Alexander MacKenzie Myles, Orlando Silvestro, Domenica Ruggeri, Giuseppina Tiziana Russo, Giovanni Squadrito, Gabriella Martino

**Affiliations:** 1 Department of Biomedical and Dental Sciences and Morphofunctional Imaging, University of Messina, 98124 Messina, Italy; 2 Department of Experimental Psychology, University of Oxford, Oxfordshire OX2 6GG, UK; liam.a.myles@outlook.com; 3 Department of Health Science, University Magna Graecia of Catanzaro, 88100 Catanzaro, Italy; orlando.silvestro@unicz.it; 4 Internal Medicine and Diabetology Unit, University Hospital of Messina, 98124 Messina, Italy; domenica.ruggeri@polime.it; 5 Department of Clinical and Experimental Medicine, University of Messina, 98124 Messina, Italy; giuseppina.russo@unime.it (G.T.R.); giovanni.squadrito@unime.it (G.S.); martinog@unime.it (G.M.)

**Keywords:** alexithymia, clinical psychology, chronic disease, psychopathology, type 1 diabetes mellitus, T1DM

## Abstract

Background: Alexithymia has been recognised as a predictor of negative outcomes in various chronic conditions, including type 2 diabetes mellitus (T2DM). However, evidence concerning its role in type 1 diabetes mellitus (T1DM) remains limited. This systematic review aims to explore the relationship between alexithymia and T1DM. Methods: In June 2025, following PRISMA guidelines, a systematic review was conducted using Scopus, PubMed, and Web of Science databases. Studies specifically addressing the relationship between alexithymia and type 1 diabetes mellitus were analysed. The search strategy included the keywords “Alexithymia” AND (“Type 1 Diabetes Mellitus” OR “T1DM”). The NIH Study Quality Assessment Tool was employed to evaluate the methodological quality of the selected studies. Results: Fifteen studies met the inclusion criteria. The systematic analysis of the literature highlighted three dominant themes: alexithymia was found to be associated with patients’ health status concerning weight and obesity, glycaemic control, and psychopathological symptoms. Moreover, alexithymia emerged as a potential predictor of adverse outcomes in T1DM self-management. Conclusions: Available evidence suggests that alexithymia has a clinically relevant impact on the management of T1DM. However, further research involving larger samples and longitudinal designs is needed to bridge the gap with other chronic conditions. Promoting evidence-based research in this area is aligned with the need for targeted psychological assessment, specific interventions, and improved care strategies.

## 1. Introduction

Type 1 Diabetes Mellitus (T1DM) is a chronic condition resulting from the autoimmune destruction of pancreatic β-cells producing insulin, whose absolute deficiency leads to significant clinical consequences [1,2]. As the most common chronic disease in youths [3], its overall impact is substantial [4,5]. Strategies and solutions to reduce the burden and increase perceived quality of life are therefore essential [6]. The interplay between chronic conditions and psychological factors currently represents a promising field of scientific investigation [7,8,9,10,11,12,13,14,15,16]. Identifying specific psychological mechanisms that worsen patients’ health and hinder disease management constitutes a significant advance in clinical research.

Notably, reduced quality of life has been widely reported in patients with T1DM [17,18,19], although the underlying psychological contributors remain insufficiently explored. Psychological functioning and health-related quality of life have emerged as key facets for T1DM [20,21,22]. These elements are identified as influencing disease course and management, often representing a serious risk for patients [20]. It is well established that psychopathology emphasises patients’ experience as both a predictor and a consequence of type 1 diabetes mellitus (T1DM) [23,24,25]. A strong link has been observed between psychological distress, general psychopathological dimensions, and diabetes, although most studies refer to Type 2 Diabetes Mellitus (T2DM) [26,27,28,29,30,31,32,33,34,35,36]. However, specific contributions directly related to T1DM remain limited.

The consequences of such psychological factors are especially evident in maladaptive responses that further complicate patients’ adjustment to the disease. Obesity and weight difficulties are strongly related to psychological functioning [37,38,39,40,41,42,43]. Lesions, insulin resistance and negative outcomes directly deriving from hyperglycaemia in obese patients with T1DM represent a major clinical concern. In this regard, glycaemic control represents a fundamental factor, and psychological influences on it must be carefully monitored [44,45,46].

Among the most influential factors for chronic conditions, alexithymia is gaining great attention. Alexithymia can be defined as difficulties or impossibility in identifying and describing affective manifestations such as emotions, feelings and mood correlates [47,48,49,50]. Its relationship with obesity, psychopathology and glycaemic control was evidenced with greater reference to T2DM [27,51,52]. A substantial body of evidence has demonstrated the impact of alexithymia on physical and psychological functioning across diverse clinical contexts, providing a solid scientific basis for its relevance in diagnosis and treatment [53,54,55,56,57,58,59,60,61,62]. The origins of the study of the impact of alexithymia on physical health are not recent [63]. A substantial number of contributions highlighted its role in terms of somatization [64,65]. In particular, it is known that phenomena linked to the amplification of bodily sensations with consequent variation in physiological responses to stress, the dysregulation of the autonomic nervous system with consequent increase in the risk of developing somatic disorders, play a fundamental role [66,67,68,69]. These phenomena are of particular relevance in the field of diabetes, where glycaemic variations are consistently influenced by affective functioning.

Given the clinical significance of alexithymia and the potential risks associated with its lack of assessment, a consistent analysis of the state of the art should precede future actions. This systematic review was aimed at portraying the state of the art regarding alexithymia in patients suffering from T1DM. A comprehensive understanding of emerging findings may serve as a strong foundation for future research and clinical interventions.

## 2. Materials and Methods

The present systematic review was performed in line with the Preferred Reporting Items for Systematic Review and Meta-analyses (PRISMA) guidelines provided by Liberati, Moher and colleagues [70,71] and PRISMA 2020 Checklist [72]. This systematic review was not registered in a public database such as PROSPERO. At the time of the protocol development, registration was not mandatory for submission to this Journal. Nevertheless, the review was conducted in accordance with PRISMA guidelines to ensure methodological rigour and transparency.

### 2.1. Information Sources and Search Strategy

Scopus, PubMed, and Web of Science databases were systematically searched to retrieve relevant publications in June 2025. The search query consisted of the following terms: “Type 1 Diabetes Mellitus” OR “T1DM” OR “T1D” AND “Alexithymia” (see Table 1). No time restrictions were applied in any of the included databases, while only English published research items were considered eligible.

### 2.2. Eligibility Criteria

Inclusion criteria were full-text articles fully published in English in peer-reviewed journals, confirmed diagnosis of T1DM, and use of validated and standardised psychodiagnostic instruments for the assessment of alexithymia. Exclusion criteria included conference abstracts, literature reviews of any kind, qualitative studies, case reports, studies lacking a clear report of T1DM diagnosis, studies without a transparent description of the inclusion process and studies involving mixed diagnostic groups.

### 2.3. Selection Process and Data Collection

**Figure 1:**
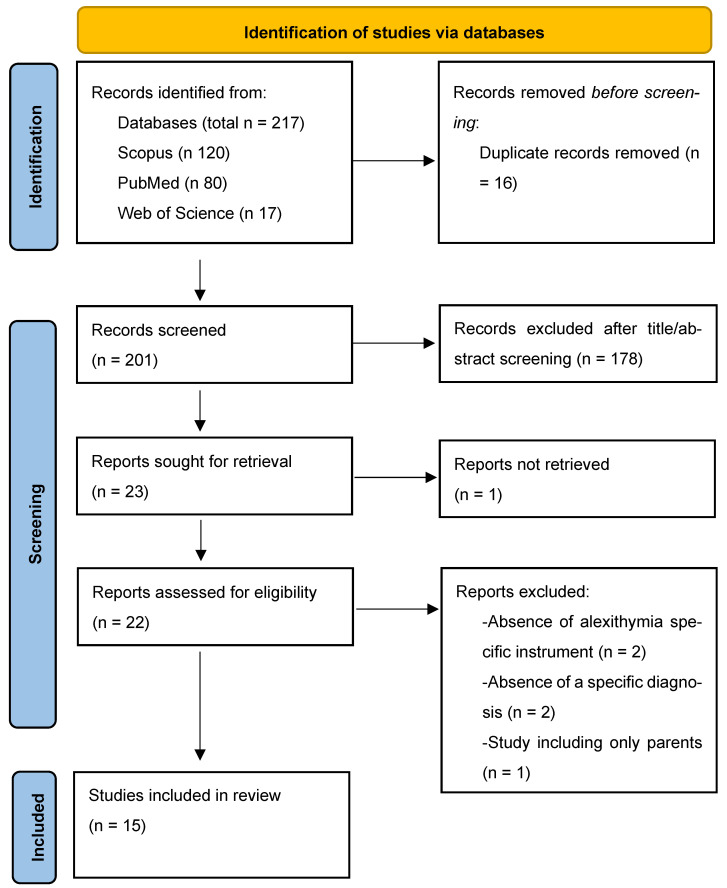
Prisma Flow Diagram.

A thorough screening process was conducted by E.M.M and G.M. before proceeding to the subsequent steps. The initial analysis allowed for the removal of duplicates. Then, titles and abstracts were examined, and only studies meeting the inclusion criteria were selected. A detailed overview of the study selection process is presented in Figure 1 (PRISMA Flow Diagram, see Results).

### 2.4. Assessment of the Methodological Quality of the Finally Extracted Studies

The NIH Study Quality Assessment Tool was employed to conduct a comprehensive evaluation of the methodological quality of the selected studies. All included studies ranked from fair to good.

## 3. Results

As reported in Figure 1, the PRISMA flow diagram highlights the results of the search strategy. In the initial step, 217 articles were identified as potentially eligible. Sixteen duplicates were removed, leaving 201 studies, which were screened based on title and abstract to assess their potential relevance. A total of 179 articles were excluded for not being pertinent to the study aim. Of the 23 remaining articles, 1 was excluded due to the unavailability of the full text. The remaining 22 reports were assessed in full, and 7 were excluded for specific methodological reasons. Ultimately, 15 studies met the inclusion criteria and are summarised in Table 2.

### 3.1. Characteristics of the Included Studies

From the analyses of the included contributions, twelve were cross-sectional studies [73,75,77,78,79,80,81,82,83,85,86,87], one was longitudinal [76] and two were case–control study [74,84]. Thirteen studies used the Toronto alexithymia scale (TAS-20) [74,76,77,78,79,80,81,82,83,84,85,86,87], one study employed the Children’s Alexithymia Measure (CAM) [73] and another one the Alexithymia Questionnaire for Children (AQC) [75]. Eleven studies included only T1DM participants [73,75,76,77,78,80,81,82,83,85,87], two studies also involved T2DM [79,84], two studies involved healthy controls [74,84], while one study distinguished between T1DM and brittle T1DM patients [86].

Concerning participants’ age groups, nine studies included adult patients [74,76,77,78,79,80,81,85,86]. One study involved a wide age range (15–58 years old) including both adolescents and adults [87]. Among the remaining studies, three focused specifically on adolescents [82,83,84] and two investigated children [73,75].

### 3.2. Weight and Risk for Obesity

Weight control, obesity risk, and related complications are increasingly recognised as Key issues in the clinical management of T1DM, since obesity is nowadays a frequent finding in T1DM patients, although with some sex- and age-related differences. Five included studies addressed this important theme. Considering the high value of studying the relationship between physical status and alexithymia, Ahmed and colleagues [73] recently highlighted the predictive role of IBM on alexithymia. Sleep disturbances were present in 59.3% of the patients, and a significant relationship emerged between overweight status and sleep disorders. Thus, a diminished quality of life was associated with higher obesity rates. Although the association between BMI and alexithymia was not directly analysed, the co-occurrence of obesity and emotional processing difficulties in the sleep-disturbed subgroup represents a noteworthy relationship that warrants attention.

The risks associated with obesity are well recognised in the scientific community. According to Melin and colleagues [78], the prevalence of abdominal overweight was 17%, with a considerable presence of physical inactivity and alexithymia directly associated. The subdimension Difficulty Identifying Feelings (DIF) was significantly associated with abdominal obesity (*p* = 0.011), and in a gender-stratified model, DIF remained statistically significant among male participants (*p* = 0.004). In female patients, abdominal obesity was associated with antidepressant use (*p* = 0.022) and reduced physical activity (*p* = 0.037). These findings suggest an indirect pathway linking alexithymia to cardiovascular complications, mediated by abdominal adiposity, and highlight the need for further investigation using longitudinal models. According to a subsequent study Melin and colleagues [80], the prevalence of abdominal obesity was triple in women. the prevalence of abdominal obesity in women was three times higher than in men (24% vs. 8%, *p* = 0.002). Alexithymia was associated with abdominal obesity in men (*p* = 0.041), further supporting its role as a relevant psychological risk factor. Moreover, according to previous results [77], and confirmed by recent data [81] the association between obesity and alexithymia appears consistent. Consequently, addressing alexithymia and related emotional dysregulation may contribute to reducing the risk of metabolic and cardiovascular complications in patients with T1DM.

### 3.3. Glycaemic Control

Glycaemic control is a key component in the management of T1DM, as it plays a central role in preventing negative outcomes. According to Chatzi and colleagues [74], alexithymia was more prevalent in T1DM patients (22%) compared to healthy controls (7.6%), even after adjusting for depressive symptoms. Higher alexithymia scores were positively correlated with illness duration and negatively with treatment intensification. Although no direct relationship with glycaemic control was observed, these findings suggest that alexithymia may influence glycaemic control through behavioural pathways, affecting disease management. According to Housiaux and colleagues [75], alexithymia represents a concrete risk for poor glycaemic control, with the subdimension Difficulty Describing Feelings (DDF) identified as a significant predictor of elevated HbA1c levels (*β* = 0.34, *p* = 0.01). Screening for alexithymia in children may provide a reliable basis for early preventive strategies. In adult patients, Luminet and colleagues [76] identified DDF as a significant predictor of poor glucose control, with higher scores associated with elevated glycated haemoglobin at admission and greater HbA1 reduction following treatment. These effects were consistent across both type 1 and type 2 diabetes, and the predictive value of DDF exceeded that of anxiety and depression, further confirming the role of alexithymia in glycaemic regulation and disease progression. A cross-sectional study also indicated an indirect link between alexithymia and glycaemic control, mediated by its association with depressive symptoms, which were independently related to elevated HbA1c levels [77]. T1DM patients with alexithymia were identified by Mnif and colleagues [84] as more exposed to fasting blood sugar than non-alexithymic ones (*p* = 0.021). These findings are further supported by evidence from adolescent populations. Shayeghian and collaborators [87] observed that DIF significantly predicted higher HbA1c levels in girls with T1DM (β = 0.15, *p* = 0.03), confirming a gender-specific vulnerability. This study also suggested that alexithymia affect self-care activities, underscoring its detrimental role in diabetes management. Measuring psychological traits and states would increase interventions efficiency and patients’ quality of life.

### 3.4. General Psychopathology

Psychopathology can be defined as a highly prevalent and heterogeneous set of factors resulting from altered mental functioning. Its role in affecting subjects’ quality of life is widely recognised. In the context of T1DM, several studies highlighted its impact, particularly concerning the predictive role of alexithymia.

According to Mellin et al. [79], T1DM patients were predominantly affected by melancholic depression, with a greater risk of misdiagnosis among alexithymic individuals. The associations between anxiety, depression and alexithymia were described as clinically significant. In a subsequent study [81], the authors reported that depression rates were higher among alexithymic patients, thus confusing the association between depression and alexithymia. A recent study by Merlo and colleagues [82] reported high rates of anxiety, depression and somatic symptoms, with alexithymia strongly linked not only to these conditions but also to intolerance of uncertainty. Building on these findings, a subsequent study confirmed their presence and further indicated that age, gender and education significantly predicted both alexithymia and intolerance to uncertainty [83]. Mnif and collaborators [84] observed that T1DM patients were more frequently alexithymic than those with T2DM. Moreover, in T1DM patients, erectile dysfunction, a common complication of diabetes, was associated with Difficulties Identifying Feelings (DIF). Finally, depression was found to be a predictor of alexithymia.

The study by Naito and colleagues [85] was the only one to examine personality traits alongside alexithymia. Their findings showed that these psychological factors negatively affected awareness of hypoglycaemia in adult T1DM patients. Cognitive barriers, including alexithymia and perfectionism, were identified as contributing factors, and maladaptive health beliefs emerged as threats to both disease progression and effective diabetes management. In line with these results, greater attention should be given to the role of psychopathology and its clear associations with alexithymia in patients with T1DM.

## 4. Discussion

T1DM is among the most challenging chronic conditions to manage on a daily basis. Constant blood sugar monitoring, fear of hyperglycemia and hypoglycemia, multiple insulin administrations, fear of the potentially debilitating complications, social stigma, and daily life obstacles are just some of the difficulties faced by patients living with T1DM and their families.

This systematic review identified several key psychological and behavioural factors which significantly influence the course of T1DM and the patient’s perceived quality of life. Compared to the abundant literature on T2DM, relatively few studies have examined psychological factors specifically affecting the T1DM trajectory. Thus, analysing the current state of the art to identify influential psychological variables is both timely and necessary. The difficulties associated with T1DM are wide-ranging, covering factors from metabolic regulation to emotional well-being. Among these, diagnostic delays and the failure to consider patients’ psychological functioning represent major clinical risks that may compromise both adaptation and disease management.

Obesity may act both as a risk factor for diabetes onset and as an aggravating element in pre-existing conditions [38,88,89,90,91,92]. However, in the case of T1DM, specific pathophysiological mechanisms must be considered [1,93]. Identifying individual psychological predictors, such as alexithymia, and their associations with other mental health dimensions could represent a significant advancement. The relationship between alexithymia and obesity has been documented [94,95,96], but the case of T1DM is qualitatively distinct due to its intrinsic risks.

According to the evidence presented, physical inactivity, sleep disturbances, direct associations between alexithymia and abdominal obesity, and the predictive value of alexithymia constitutes robust findings. In this context, interventions should be preceded by validated assessment procedures. Given the potentially severe outcomes of T1DM, this interrelated risk factor cannot be overlooked. The connections observed reflect well-established research domains [55,97,98] that must be specifically contextualised within T1DM.

Glycaemic control also emerges as a central determinant in the clinical course of T1DM [99,100,101]. In this domain as well, alexithymia represents a serious concern. Its associations and predictive value are consistent with current findings in the literature [45,102]. While emotional distress has recently been considered a treatment target in T1DM [103], alexithymia remains underexplored. Notably, studies specifically centred on the role of alexithymia in T1DM are lacking.

Alexithymia has been identified as a precursor of maladaptive beliefs, as confirmed by a recent systematic review and meta-analysis [104], although no direct reference to diabetes was made. This highlights the need for future studies that include this clinical population. As previously noted, eating behaviours play a critical role, and conditions such as diabulimia have been recognised as highly influential [105]. Given the impact of alexithymia on individuals’ health status and quality of life [55,57], together with the importance of glycaemic control, these factors must be systematically addressed.

Although alexithymia is not classified as a form of psychopathology, its associations with psychiatric conditions are well documented [106,107,108,109]. According to the findings of this review, a clear link between psychopathology and alexithymia was evident. Given the impact of affective disorders on diabetic patients and their well-being [110,111], closer clinical attention is warranted. Moreover, the risk of missed diagnosis due to alexithymia and its influence on illness-related beliefs has emerged as a relevant issue. This is consistent with the difficulty that alexithymic individuals have in identifying and describing affective states.

As a psychological vulnerability factor [56,112,113], alexithymia may contribute to the onset of psychopathology and worsen existing illness conditions. Conversely, the chronic nature of T1DM can itself trigger reactive psychopathological responses, leading to decreased quality of life and the emergence of typical clinical features. Somatisation disorders may also arise as a direct expression of alexithymic functioning. However, aside from the studies included in this review, data specifically addressing T1DM are still lacking. Therefore, psychopathology should be understood as a multifaceted construct, both influenced by and influencing alexithymia, with important implications for clinical outcomes. As such, clinical pathways must not ignore their presence and potential impact.

T1DM is a serious chronic illness with potentially severe consequences. The fact that most existing research on alexithymia focuses on T2DM represents a major limitation in the current scientific literature. The psychological dimensions of T1DM remain significantly underexplored. In light of what has emerged, alexithymia must not remain a neglected factor. Although it is still necessary to study its impact in the field of T1DM, the results obtained from chronic diseases must serve as a solid basis for further research. In particular, when it comes to chronic conditions that accompany the patient throughout life, the role of these psychological factors is fundamental. As previously reported, pathological outcomes linked to poor management of the disease can be serious [114,115]. In this case, affective dysregulation, the use of inadequate defences and coping has a profound impact on adherence to treatments. In addition to the scarcity of studies, there is a clear need for research designs capable of capturing the dynamic evolution of psychological and physical factors over time.

Through this systematic review, it was possible to highlight these shortcomings and the limitations of the current body of evidence. However, acknowledging these gaps offers a valuable opportunity to advance research and improve knowledge in the field of T1DM.

## 5. Strengths and Limitations

This review was aimed at portraying the relationship between alexithymia and T1DM. Based on the findings, alexithymia emerges as a clinically relevant risk factor that should be systematically assessed to prevent negative outcomes. However, this review also presents several limitations.

Although all included studies employed quantitative methodologies, the majority adopted a cross-sectional design. The lack of longitudinal data and the limited capacity to detect changes over time, especially in response to psychological or behavioural interventions, may reduce the interpretability and clinical generalisability of the results. Furthermore, considerable heterogeneity was observed in the age groups of participants, ranging from childhood to adulthood. This demographic variability may limit the comparability of findings across studies, although it also highlights the potential burden of alexithymia across different stages of life.

Most of the studies utilised the same assessment instrument for alexithymia, which, while contributing to methodological consistency, limited the possibility of convergent validation with alternative measures. Furthermore, all included studies’ design was quantitative. Qualitative research considering subjective experience could have enriched the panorama. Additionally, the reliance on self-report questionnaires may represent a weakness, as these instruments are susceptible to reporting biases and may not fully capture the clinical complexity of emotional dysregulation. Thus, since most of the results emerged in European contexts, this may limit generalisability.

Despite these limitations, to the best of our knowledge, this is the first systematic review specifically focused on the role of alexithymia in the context of T1DM. The substantial gap in the literature that motivated this review should be addressed through rigorous, longitudinal research capable of generating evidence-based insights and guiding future clinical practice.

## 6. Conclusions

Through this systematic review, relevant evidence emerged supporting the association between alexithymia and T1DM. The main domains identified were obesity and weight-related difficulties, psychopathology, and glycaemic control.

About obesity, alexithymia was found to influence patients’ weight status, emerging as a potential predictor of obesity. Given the specific metabolic and behavioural dynamics of T1DM, this association raises particular concern.

In the area of psychopathology, alexithymia showed strong associations with anxiety, depression, and somatisation. Across most of the included studies, a clear relationship was observed between alexithymia and general psychopathological burden. Beyond its predictive value, alexithymia was shown to negatively affect patients’ psychological adjustment, overall health status, and the effectiveness of disease management.

Furthermore, glycaemic control, crucial for the long-term prognosis of T1DM, was consistently reported as being adversely influenced by alexithymia. This suggests that alexithymia may represent an emotional barrier to effective diabetes self-management.

These findings should inform clinical practice, encouraging the routine assessment of alexithymia and the implementation of targeted psychological interventions aimed at mitigating its impact on T1DM outcomes.

## Figures and Tables

**Table 1:** List of terms entered in the database search.

Number	Term
1	ALEXITHYMI * [all fields]
2	ALEXITHYMIA [all fields]
3	TYPE 1 DIABETES MELLITUS [all fields]
4	T1DM [all fields]
5	T1D [all fields]
6	2 AND 3 OR 4 OR 5
7	1 AND 6

*: Asterisk, truncation.

**Table 2:** Included studies and main characteristics.

Authors	Year	Study Design	State	Sample	Alexithymia Measure	Findings	NIH Study Quality Assessment
Ahmed et al. [73]	2024	Cross-sectional	Egypt	118 T1DM patients	Children’s Alexithymia Measure (CAM)	Alexithymia was associated with sleep difficulties. Higher alexithymia scores (β = 0.187, *p* = 0.033) and elevated BMI (β = 0.257, *p* = 0.005) independently predicted sleep difficulties. CAM scores were higher in subjects suffering from sleep disorders.	Good
Chatzi et al. [74]	2009	Case–control study	Greece	96 T1DM patients and 105 health controls	Toronto Alexithymia Scale (TAS-20).	Higher rates of alexithymia were found in T1DM patients (22.2% vs. 7.6%). Trend-level associations were observed between alexithymia and longer diabetes duration, as well as reduced treatment intensity.	Fair
Housiaux et al. [75]	2010	Cross-sectional	Belgium	45 T1DM patients	Alexithymia Questionnaire for Children (AQC)	Higher DDF score significantly predict poorer glycaemic control in children with T1DM (β = 0.34; *p* = 0.01), accounting 12% of variance. Alexithymia was also associated with disease severity, even though further studies were necessary to confirm data.	Good
Luminet et al. [76]	2006	Longitudinal study	Belgium	64 T1DM patients	Toronto Alexithymia Scale (TAS-20)	Difficulty describing feelings (DDF) predicted lower glucose control. Its role overcomes the predictive power of anxiety and depression. Data were stable along T1DM and T2DM, configuring DDF as a strong predictor worsening disease. Alexithymia total score indexes changed between T1 and T2 (46.44 ± 10.86 vs. 35.64 ± 14.03).	Good
Melin et al. [77]	2013	Cross-sectional study	Sweden	292 T1DM patients	Toronto Alexithymia Scale (TAS-20)	Alexithymia was associated with lower glycaemic control and depression in bivariate analyses. Only symptoms of depression independently predicted glycaemic control in T1DM patients (AOR = 4.8, *p* = 0.001; AOR = 19.8, *p* < 0.001 in women).	Good
Melin et al. [78]	2017	Cross-sectional study	Sweden	284 T1DM patients	Toronto Alexithymia Scale (TAS-20)	Alexithymia and its subdimension DIF were associated with abdominal obesity (*p* = 0.028; *p* = 0.011). Stratified analysis revealed that the association between difficulty identifying feelings and central adiposity was notably stronger in male patients (*p* = 0.004). In women, abdominal obesity was linked to antidepressant use and physical inactivity (*p* = 0.022 and *p* = 0.037).	Good
Melin et al. [79]	2017	Cross-sectional study	Sweden	148 T1DM patients and 24 T2DM patients	Toronto Alexithymia Scale (TAS-20)	T1DM and T2DM differed in terms of alexithymia associations with depression and anxiety. Alexithymia was associated with depression in T2DM patients (67% vs. 11%), while T1DM patients presented lower scores of alexithymia (47% vs. 11%).	Good
Melin et al. [80]	2019	Cross-sectional study	Sweden	190 T1DM patients	Toronto Alexithymia Scale (TAS-20)	Alexithymia was associated with abdominal obesity in men (*p* = 0.018). As a concrete risk factor, alexithymia was the only demonstrated feature strongly influencing abdominal obesity in men with T1DM.	Good
Melin et al. [81]	2021	Cross-sectional study	Sweden	292 T1DM patients	Toronto Alexithymia Scale (TAS-20)	T1DM patients with alexithymia (15%) had 5.7 times higher prevalence of depression, twice as high prevalence of anxiety, 1.9 times higher prevalence of abdominal obesity, and 3.2 times higher prevalence of combined anxiety and abdominal obesity compared to patients without alexithymia. Elevated levels of Galectin-3 Binding Protein (Gal3BP) were also 2.3 times more frequent among alexithymic patients.	Good
Merlo et al. [82]	2024	Cross-sectional study	Italy	137 T1DM patients	Toronto Alexithymia Scale (TAS-20)	Alexithymia emerged as significant for T1DM patients (total score = 53.766 ± 11.907). Alexithymia was associated with different extents to depression, anxiety, obsession eating disorders and somatisation. Thus, a clear positive relationship emerged between alexithymia and intolerance to uncertainty. Moreover, age predicted greater rates of externally oriented thinking, and female gender predicted greater difficulty identifying feelings.	Good
Merlo et al. [83]	2024	Cross-sectional study	Italy	105 T1DM patients	Toronto Alexithymia Scale (TAS-20)	A concerning presence of alexithymia was highlighted in T1DM patients (total score = 54.67 ± 12.35). Age, education and illness duration were negatively associated with psychopathology. Alexithymia and intolerance to uncertainty were positively associated. Externally oriented thinking and inhibitory anxiety significantly differed among male and female patients aged 14 to 18 years, with higher scores in male subjects.	Good
Mnif et al. [84]	2014	Cross-sectional study	Tunisia	50 T1DM patients, 75 T2DM patients and 122 healthy controls.	Toronto Alexithymia Scale (TAS-20)	T1DM showed higher alexithymia prevalence compared to healthy controls (46% vs. 21.5%). Alexithymic patients had significantly higher fasting glucose levels and a greater incidence of complications (21.7% vs. 14.8%). Erectile dysfunction was associated with DIF (*p* = 0.012), while EOT was linked to irregular follow-up and poor treatment adherence (*p* = 0.032). Depression emerged as significant predictor of alexithymia, which also showed positive correlations with anxiety (*p* = 0.008) and depression scores (*p* = 0.003).	Fair
Naito et al. [85]	2021	Cross-sectional study	United Kingdom	90 T1DM patients	Toronto Alexithymia Scale (TAS-20)	Alexithymia positively correlated with cognitive barriers related to Hyperglycaemia Avoidance Prioritised and Asymptomatic Hypoglycaemia Normalised. High alexithymia scores were found in patients with impaired awareness of hypoglycaemia (17.6% vs. 1.9%). These findings highlight alexithymia as a psychological trait linked to risk factors for hypoglycaemia and impaired self-management.	Good
Pelizza & Pupo [86]	2019	Cross-sectional study	Italy	44 Brittle T1DM and 88 T1DM patients	Toronto Alexithymia Scale (TAS-20)	Patients suffering from a brittle form of T1DM were more alexithymic than controls (18.2% vs. 2.3%). Alexithymia was significantly associated with anxiety, obsession, depression, paranoid ideation, somatisation and psychoticism. Obsessive–compulsive traits (β = 0.44, t = 3.19, *p* < 0.01) and somatisation (β = 0.59, t = 4.76, *p* < 0.05) emerged as independent predictors of alexithymia.	Good
Shayeghian et al. [87]	2020	Cross-sectional study	Iran	150 T1DM patients	Toronto Alexithymia Scale (TAS-20)	Alexithymia, and, in particular, DIF predicted poorer disease management in both female (β = −0.04, *p* = 0.02; β = −0.06, *p* = 0.04) and male patients (β = −0.07, *p* = 0.01; β = −0.11, *p* = 0.01). DDF predicted poorer self-management only in males (β = −0.16, *p* = 0.02), while in females, alexithymia and DIF were associated with HbA1c levels (β = 0.15, *p* = 0.03). Alexithymia can affect self-management for both male and female subjects suffering from T1DM.	Good

Note. AOR: Adjusted odds ratio; AQC: Alexithymia Questionnaire for Children; BMI: Body Mass Index; CAM: Children’s Alexithymia Measure; DDF: Difficulty describing feelings; DIF: Difficulty identifying feelings; EOT: Externally oriented thinking; Gal3BP: Galectin-3 Binding Protein; HbA1c: Haemoglobin A1c; T1DM: Type 1 Diabetes Mellitus; T2DM: Type 2 Diabetes Mellitus; TAS-20: Toronto Alexithymia Scale.

## Data Availability

No new data were created or analyzed in this study.

## Abbreviations

The following abbreviations are used in this manuscript:

T2DM	Type 2 Diabetes Mellitus
T1DM	Type 1 Diabetes Mellitus
T1M	Type 1 Diabetes
TAS-20	Toronto Alexithymia Scale-20
DIF	Difficulty Identifying Feelings
DDF	Difficulty Describing Feelings
EOT	Externally Oriented Thinking
CAM	Children’s Alexithymia Scale
AQC	Alexithymia Questionnaire for Children
HbA1	Haemoglobin A1
HbA1c	Haemoglobin A1c
